# Link Between Antibiotic Persistence and Antibiotic Resistance in Bacterial Pathogens

**DOI:** 10.3389/fcimb.2022.900848

**Published:** 2022-07-19

**Authors:** Wolfgang Eisenreich, Thomas Rudel, Jürgen Heesemann, Werner Goebel

**Affiliations:** ^1^ Bavarian NMR Center – Structural Membrane Biochemistry, Department of Chemistry, Technische Universität München, Garching, Germany; ^2^ Chair of Microbiology, Biocenter, University of Würzburg, Würzburg, Germany; ^3^ Max von Pettenkofer-Institute, Ludwig Maximilian University of Munich, München, Germany

**Keywords:** persistence, resistance, ATP-DnaA complex, DNA replication initiation, bacterial pathogens

## Abstract

Both, antibiotic persistence and antibiotic resistance characterize phenotypes of survival in which a bacterial cell becomes insensitive to one (or even) more antibiotic(s). However, the molecular basis for these two antibiotic-tolerant phenotypes is fundamentally different. Whereas antibiotic resistance is genetically determined and hence represents a rather stable phenotype, antibiotic persistence marks a transient physiological state triggered by various stress-inducing conditions that switches back to the original antibiotic sensitive state once the environmental situation improves. The molecular basics of antibiotic resistance are in principle well understood. This is not the case for antibiotic persistence. Under all culture conditions, there is a stochastically formed, subpopulation of persister cells in bacterial populations, the size of which depends on the culture conditions. The proportion of persisters in a bacterial population increases under different stress conditions, including treatment with bactericidal antibiotics (BCAs). Various models have been proposed to explain the formation of persistence in bacteria. We recently hypothesized that all physiological culture conditions leading to persistence converge in the inability of the bacteria to re-initiate a new round of DNA replication caused by an insufficient level of the initiator complex ATP-DnaA and hence by the lack of formation of a functional orisome. Here, we extend this hypothesis by proposing that in this persistence state the bacteria become more susceptible to mutation-based antibiotic resistance provided they are equipped with error-prone DNA repair functions. This is - in our opinion - in particular the case when such bacterial populations are exposed to BCAs.

## 1 Introduction

### 1.1 Antibiotic Resistance Acquired by Mutations and Horizontal Gene Transfer

Human bacterial pathogens with increased resistance to one or even more antibiotics represent a severe world-wide health problem. Antibiotic resistance, in contrast to the antibiotic-tolerant persistence phenotype (discussed below) is always genetically determined by well-defined genes.

Resistance genes providing defense to the detrimental action of their own dangerous drugs, are found in most antibiotic-producing microorganisms or evolve in natural environments by the interaction between antibiotic producers and the surrounding antibiotic-sensitive bacteria (Aminov and Mackie, 2007; Wright, 2007; Wright, 2010; Martinez, 2014; Peterson and Kaur, 2018; Larsson and Flach, 2021). Most of the known antibiotics are produced by different soil bacteria, especially members of the genera *Streptomyces* and *Bacillus*, and by some fungi, especially members of the genera *Penicillium* and *Cephalosporium*. Production of antibiotics may help the producers to limit the competition by other antibiotic-sensitive microorganisms in their immediate environment (Demain and Fang, 2000; Falkinham et al., 2009; Foster and Woodruff, 2010). In response, some of these latter co-resident bacteria acquire or evolve genes coding for a variety of biochemical mechanisms that protect them from the killing or growth-inhibiting actions triggered by these antibiotics, thus generating a stable genetically determined resistance to one or even more antibiotics. These antibiotic resistance (ABR) genes are passed on to their next generations and may also be eventually transferred horizontally to other bacteria in their immediate environment.

Human bacterial pathogens can acquire resistance to antibiotics by two major genetic strategies which protect the pathogens from the detrimental action of antibiotics when exposed to them: (i) by specific mutations and the passing on of the mutation-based ABR to the offspring by vertical gene transfer (VGT) and (ii) by acquisition of external ABR genes through horizontal gene transfer (HGT). For details see (Foster, 2007).

In short, chromosomal mutations resulting in ABR (i) lead to modified cell targets which in general can no longer bind the respective antibiotics, (ii) decrease the uptake efficiency of the bacterial cell for the antibiotic, (iii) increase the activity of specific efflux pumps causing extrusion of the antibiotic from the bacterial cell, (iv) inactivate repressors for the expression of genes that code for enzymes (especially specific ß-lactamases) which inactivate the antibiotic (in this case ß-lactam antibiotics) (Alekshun and Levy, 2007; Blair et al., 2015; Munita and Arias, 2016; Li et al., 2019a), or (v) extend the hydrolytic activity towards a broad spectrum of ß-lactam antibiotics (Bradford, 2001).

A large number of mutations in chromosomal genes has been identified that lead to resistance of many bacterial pathogens to one or even more antibiotics (belonging to most classes of antibiotics). Acquisition of mutation-based ABR is quite diverse and varies significantly among bacterial pathogens (George, 1996; Davies, 1997; Martinez and Baquero, 2000; Zankari et al., 2012; Lopez-Causape et al., 2018). This “mutational antibiotic resistance” arising by treatment with BCAs (and other antibiotics) will be described below in more detail.

HGT of ABR genes within and between bacterial species is achieved essentially by three major genetic mechanisms: transformation, transduction and conjugation (Burmeister, 2015; Blokesch, 2016; Zhou et al., 2021). Transformation includes the transfer of naked DNA to competent (Com) recipient bacteria and its incorporation into the recipient´s chromosome by homologous recombination. Transduction uses bacteriophages as vehicles for the DNA transfer and has been reported to play a role in the microbiome of cystic fibrosis patients, in particular of *Pseudomonas aeruginosa* (Rolain et al., 2011). Conjugation requires the direct contact between donor and recipient bacteria, often achieved with the help of specific cell appendages/pili. DNA transfer by conjugation most frequently involves mobile genetic elements such as plasmids, transposons and integrons (Babakhani and Oloomi, 2018; Partridge et al., 2018). However, transfer of chromosomal DNA from the donor to the recipient may also occur by conjugation (Manson et al., 2010). A recently discovered fourth horizontal transfer mechanism of plasmids *via* extracellular vesicles (EVs, vesiduction) shedded from the outer membrane has been described for *Acinetobacter baumannii* and *Escherichia coli* (Rumbo et al., 2011; Tran and Boedicker, 2017; Toyofuku et al., 2019). However, the impact of EV-mediated plasmid transfer for evolution of ABR is still unclear.

For plasmid transfer by conjugation the donor requires sufficient ATP and proton motive force (PMF) for the involved type IV secretion system (T4SS) and the recipient must be able to produce immediately double-stranded DNA (Palmen et al., 1994). For transformation, the recipient must also be energized (ATP; PMF) to induce competence (Com) and the DNA uptake machinery (Domenech et al., 2020; Rosch and Tuomanen, 2020). However, it is unclear if vesiduction requires energy for plasmid transfer.

Among human bacterial pathogens, natural transformation has been shown as the major HGT mechanism for the transfer of antibiotic resistance genes for *Streptococcus pneumoniae*, *Helicobacter pylori*, *Campylobacter jejuni*, *Neisseria gonorrhoeae*, and *Acetinobacter* spp. (Domingues et al., 2012). Transduction appears to be the most common HGT mechanism for transfer of ABR (and virulence) genes in *Staphylococcus aureus* (Chen and Novick, 2009; Varga et al., 2012).

Conjugation is probably the most efficient HGT mechanism to transfer ABR genes to clinically important bacterial pathogens and plays the most relevant role in the dissemination of ABR (Thomas and Nielsen, 2005; Munita and Arias, 2016). Whereas transformation and transduction normally depend on homologous recombination and DNA repair which restricts successful DNA transfer to related bacteria with sufficient homology between donor and recipient genomes, conjugation allows DNA transfer (including ABR genes) without homologous recombination. Especially broad-host-range plasmids allow the spreading of ABR genes from a donor bacterium even to distantly related recipient bacteria (Thomas and Nielsen, 2005; Johnston et al., 2014).

ABR genes are often assembled in integrons, containing genes and genetic structures which facilitate the capture and mobilization of foreign genes by site-specific recombination and thereby the risk of their spreading (White et al., 2001; Rowe-Magnus et al., 2002; Stalder et al., 2012). Integrons often carry multiple ABR genes which are localized on mobile genetic elements, such as plasmids and transposons. These genetic structures strongly contribute to the spreading of multiple ABR in the environment and in clinical context (Jones et al., 1997; Fluit and Schmitz, 2004; Randall et al., 2004; Kaushik et al., 2019).

### 1.2 Conditions and Proposed Mechanisms Leading to Antibiotic-Tolerant States (Persistence and Heteroresistance)

Whereas ABR in bacteria is in general a rather stable, genetically determined state, antibiotic persistence and heteroresistance confer transient tolerance to (often multiple) antibiotics. Both antibiotic-tolerant phenotypes occur temporarily in subpopulations of growing and/or resting bacterial populations. For definition of antibiotic persistence and heteroresistance, see (Balaban et al., 2019).

**Figure 1:**
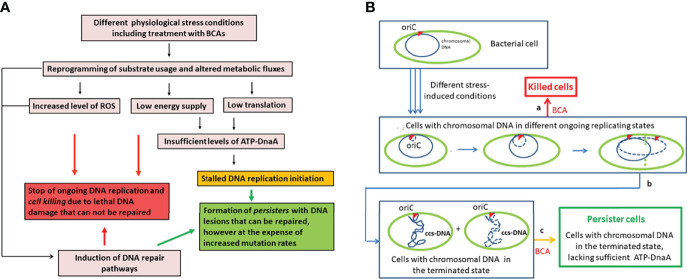
Formation of the antibiotic-tolerant persistence state (according to our hypothesis). **(A)** Different stress-induced physiological conditions (including treatment with BCAs) lead to increased production of reactive oxygen species (ROS) that damage cell components including DNA, to reduced energy production that inhibits DNA replication and repair, and to reduced metabolic activities. **(B)** Replicating DNA is particularly sensitive to irreparable damage (especially double strand breaks) which leads to killing of the bacteria **(a)**. Cells with terminated chromosomal DNA replication but unable to re-initiate replication (due to the lack of sufficient ATP-DnaA initiator complex – see text for details) have closed circular supercoiled (ccs) DNA that is rather insensitive to lethal DNA damage **(b)**. According to our hypothesis, this cellular state arrested in the termination of DNA replication represents the state of persistence **(c)**. Solid blue circle, parental chromosomal DNA; dashed blue circle, replicating DNA; dashed green vertical line, beginning of cell division; ccs, closed circular supercoiled DNA in the terminated state; oriC, origin of replication marked by the red triangle; BCAs, bactericidal antibiotics.

In contrast to ABR populations, the antibiotic-persistent subpopulations do not or hardly grow in the presence of the antibiotic(s) and remain genetically unaltered compared to the antibiotic-sensitive majority of the bacterial populations from which they arise. The persister subpopulations differ significantly in size, strongly depending on the environmental conditions of the respective bacterial cultures. Antibiotic-persistent subpopulations become again sensitive to the antibiotic(s) upon resuscitation under favorable growth conditions and in the absence of the antibiotic(s) (Gollan et al., 2019; Jung et al., 2019; Bakkeren et al., 2020). Some of the main features of antibiotic persistence are summarized in **Figure 1B**.

Heteroresistance describes a phenotype in which a subpopulation of cells in a bacterial population shows – in the presence of an antibiotic – a significant reduction in antibiotic susceptibility compared to the main population. In contrast to an antibiotic-persistent subpopulation, a heteroresistant subpopulation is – similar to an ABR population – still able to grow in the presence of the antibiotic. However, the heteroresistant phenotype is – in contrast to the true resistant phenotype – in general unstable and rapidly reverts to susceptibility in the absence of the antibiotic, similar to the antibiotic persistent phenotype. Heteroresistance seems to be mainly caused by the amplifications of genes that cause ABR and its transient character appears to be due to the instability of this genetic arrangement (Nicoloff et al., 2019). Heteroresistance has been observed for different bacterial species and different classes of antibiotics (Andersson et al., 2019). Clinical studies show that heteroresistance appears to be also a serious problem associated with antibiotic treatment (Band and Weiss, 2019). The heteroresistance phenotype appears to be, however, less relevant for our line of discussion and will not be further addressed in this review. For more details on this topic, see (Dewachter et al., 2019; Gollan et al., 2019; Jung et al., 2019; Bakkeren et al., 2020).

#### 1.2.1 A Short Summary of the Described Mechanisms Leading to Persistence in Bacteria

In the following, we will focus on the persistence phenotype. The fact that small antibiotic-persistent subpopulations are present even in bacterial populations growing in rich culture media suggests that suitable physiological state(s) leading to antibiotic-persistence can stochastically occur in few cells of a bacterial population. Various environmental and growth conditions, often causing stress on the bacterial cell, have been described that favor the generation of antibiotic-persistent subpopulations. The most extensively studied stress conditions leading to antibiotic persistence include:

(i) increased release of the toxin component of various toxin/antitoxin (TA) modules, especially those belonging to the type I and type II TA modules (Maisonneuve et al., 2011; Balaban et al., 2013; Maisonneuve and Gerdes, 2014; Cheverton et al., 2016; Gerdes, 2016; Kedzierska and Hayes, 2016; Rycroft et al., 2018),

(ii) induction of the stringent response together with the alarmone guanosine tetra-/pentaphosphate ((p)ppGpp) (Korch et al., 2003; Germain et al., 2015; Liu et al., 2017b),

(iii) induction of the RpoS-mediated general stress response (Mok et al., 2015; Liu et al., 2017b; Mok and Brynildsen, 2018),

(iv) oxidative stress, also caused by BCAs, leading to reactive oxygen species (ROS) production (Wu et al., 2012) with subsequent DNA damage and induction of the SOS response (Dörr et al., 2009; Kreuzer, 2013; Völzing and Brynildsen, 2015),

(v) impaired ATP production (Amato et al., 2013; Amato and Brynildsen, 2015; Shan et al., 2017; Mok and Brynildsen, 2018), and

(vi) feedback-regulation of cellular core processes, i.e. chromosome replication, transcription and translation (Pontes and Groisman, 2019; Pontes and Groisman, 2020), and protein aggregation (Pu et al., 2019; Bollen et al., 2021).

Recently, epigenetic regulation has been suggested to play a major role within bacterial persistence formation (Riber and Hansen, 2021) and DNA adenine methylation by Dam has been shown to affect persister formation in uropathogenic *E. coli* (Xu et al., 2021).

Triggered by corresponding environmental (stress) stimuli including BCAs, all these processes can lead to a very significant increase of an antibiotic-tolerant fraction within bacterial populations. Thus, antibiotic-tolerant persistence is not only due to reduced inhibition of the actual antibiotic targets, but is also strongly supported by a variety of altered physiological events that are caused by antibiotics, such as disturbance of cell metabolism (Yang et al., 2017; Zampieri et al., 2017) (see below, for further details), e.g. leading to decreased ROS production and hence less DNA lesions (Völzing and Brynildsen, 2015; Hong et al., 2019).

Therefore, it is not surprising that, unlike the limited number of mutations providing antibiotic resistance, a rather wide range of mutations seems to be able to influence the level of antibiotic tolerance (persister phenotype) in bacteria, as mentioned above. This fact highlights the fundamental difference between the mechanisms of tolerance (persistence) and resistance against antibiotics and raises the question of how these two mechanisms are linked.

#### 1.2.2 Metabolism and Persistence

Metabolism plays an important role in initiating, maintaining and ending the persister state in bacteria (Amato et al., 2013; Orman and Brynildsen, 2013; Amato et al., 2014; Prax and Bertram, 2014; Hartman et al., 2017; Radzikowski et al., 2017; Cabral et al., 2018). However, our knowledge on the precise metabolic events occurring in this state is still fragmentary, since the specifically altered processes of persister cells are difficult to determine due to the generally low abundance of the persister subpopulations, their transient nature and the often similar morphology of persister and normal cells (Orman et al., 2015; Rowe et al., 2016).

It is reasonable to assume that in order to survive antibiotic stress, the persister cells must shut down or silence essential physiological cell functions which antibiotics would irreversibly damage, but maintain viability during stasis, in order to resume growth once the stress is lifted. Persistence, often described as a “dormant state”, obviously represents a metabolic state of low activity (Spoering et al., 2006; Prax and Bertram, 2014; Radzikowski et al., 2016) that is characterized by a large decline of most anabolic processes, a continued low ATP production *via* specific catabolic processes (with substrate phosphorylation) and/or respiration (*via* oxidative phosphorylation) still allowing membrane energizing and repair processes (in particular DNA repair) by residual or even increased synthesis of a special set of proteins supporting these processes (Babin et al., 2017; Ayrapetyan et al., 2018). Recently, dormancy depth of bacterial persisters was related to protein aggregation in the cytosol, due to the decline of the amphiphilic ATP concentration (acting as a biological hydrotrope) in the cytosol, resulting in a state similar to that of the viable but non-culturable (VBNC) state (Patel et al., 2017; Pu et al., 2019; Bollen et al., 2021).

The persistence-promoting metabolic processes apparently occur already stochastically in untreated bacterial populations but are significantly enhanced in the presence of (especially bactericidal) antibiotics (and under the other stress conditions mentioned above). This has been especially shown by *in vitro* culture studies and also in animal models (Dörr et al., 2010; Radzikowski et al., 2016; Gutierrez et al., 2017; Meylan et al., 2017; Yang et al., 2017).

Bacterial persisters have been associated with chronic or recurrent infections (Mulcahy et al., 2010; Grant and Hung, 2013; Fisher et al., 2017), but, on the whole, the clinical significance of bacterial persistence (e.g. in chronic infections) is less clear and still under debate (Westblade et al., 2020; Moldoveanu et al., 2021).

#### 1.2.3 A Novel Hypothesis to Explain the Emergence of Bacterial Persistence

Regarding the physiological aspects linked to persistence, we would also like to refer to our recent review (Eisenreich et al., 2020). There, we focused in particular on persistence of intracellular bacterial pathogens (IBPs) and pointed out that some of these pathogens which are able to successfully reach a persistence state, in particular obligate IBPs like *Chlamydiae*, lack most of the specific stress response factors and pathways which - as stated above - apparently support the generation of the persistence state in the model bacteria that are mainly used for studying persistence., i.e. in particular *E. coli* (in particular laboratory K12 strains). Considering this fact, we proposed a new hypothesis that might explain persister formation not only for the latter IBPs but for bacteria in general (**Figure 1A**). This hypothesis is mainly based on a metabolic strategy that appears to be responsible for the replication of IBPs within mammalian host cells which we termed “bipartite metabolism” (Grubmüller et al., 2014; Eisenreich et al., 2015).

In short, bipartite metabolism consists of two distinct metabolic networks:

(i) A mainly catabolic network (previously termed P1) (Eisenreich et al., 2020) which is devoted to the production of energy and some essential TCA intermediates (e.g. oxaloacetate, α-ketoglutarate, succinate) that are substrates for other cellular processes, like cell wall synthesis or respiration. This network is fed mainly by C_3_ substrates (e.g. pyruvate, glycerol).

(ii) A largely anabolic network (previously termed P2), which is mainly fed by intermediates of the upper part of the glycolytic pathway and the pentose phosphate pathway (PPP). P2 is essential for the production of indispensable anabolic components (in particular carbohydrate components for cell envelope biosynthesis) that cannot be provided by the host cell.

The P1 and P2 networks are differently linked depending on the physiological state of the host cell (Eisenreich et al., 2017; Eisenreich et al., 2019). For the active multiplication of IBPs within host cells, the full function of both networks is necessary. In the persistent state of the IBPs, P2 will be largely turned off whereas P1 must still function, albeit at a low level, to provide sufficient energy to maintain vital cell processes, such as DNA repair and membrane energizing. Such a specific low metabolic flux is most likely the basic core for establishing persistence not only for IBPs but also for other bacteria with greater metabolic capacities, while persistence-stimulating factors, like various TA modules, ppGpp, RpoS etc. may have a more modulating and/or stabilizing function for establishing this basic metabolic background activity (Radzikowski et al., 2016).

Our hypothesis assumes that persistence represents a metabolic state in which the DNA replication is terminated, but re-initiation of DNA replication is prevented due to an insufficient amount of active ATP-DnaA initiator caused either by insufficient ATP, blockade of *de novo* DnaA protein synthesis, failure to anchor the ATP-DnaA-OriC initiation complex to the membrane site, or the combination of these events (**Figure 1**). This mechanism of persister formation could be valid for bacteria in general, even for those, like *Chlamydiae* and *Rickettsiae* that lack most of the factors and stress response pathways shown to enhance persistence in model bacteria, like *E. coli.* In contrast to the latter factors, nearly all bacteria including the obligate IBPs depend on active ATP-DnaA for re-initiating DNA replication and hence cell division (Kaguni, 2006; Leonard and Grimwade, 2015; Hansen and Atlung, 2018; Trojanowski et al., 2018). It is also interesting to note that all previously described conditions, as well as BCAs that lead to increased persister formation, inhibit either DNA replication, transcription, translation or cell envelope (peptidoglycan) synthesis and hence block either *de novo* DnaA protein synthesis, reduce the ATP level thus preventing the critical cellular concentration of the ATP-DnaA complex, or block the formation of a new anchoring site for this initiation complex at the membrane. However, all of these steps are necessary for the formation of the ATP-DnaA initiation complex at the chromosomal OriC site (origin of replication) and hence for the initiation of a new round of DNA replication (Matsunaga et al., 1986; Regev et al., 2012; Samadpour and Merrikh, 2018; Li et al., 2019b). Recently, this hypothesis was also supported by the observation that under starvation *E. coli* converts ATP to polyphosphate (PolyP)-chains by PolyP-kinase (Pkk) which serves as a matrix for Lon protease binding and activation. This results in degradation of DnaA-ADP (Gross and Konieczny, 2020; Charbon et al., 2021). In addition to DnaA-degradation, starvation also results in modulating the super-coiled state of the origin of replication (Kraemer et al., 2019). For more details on this hypothesis, see (Eisenreich et al., 2020).

However, the generation of the antibiotic-tolerant persister state (especially when generated by BCAs) does not only temporarily prevent the complete eradication of a basically antibiotic-sensitive bacterial population but may also enhance the development of mutants with genetically fixed antibiotic resistance. This is frequently observed in *Pseudomonas*-infected cystic fibrosis (CF) patients (Oliver and Mena, 2010; Levin-Reisman et al., 2019; Windels et al., 2019; Colque et al., 2020; Huemer et al., 2020; Sulaiman and Lam, 2021). In the following, we will address this connection between antibiotic persistence and antibiotic resistance on the example of BCAs also taking into account the described hypothesis that persistence is linked to a decreased level of the ATP-DnaA complex which is insufficient for re-initiation of DNA replication.

## 2 Bactericidal Antibiotics– Generation of Antibiotic Persistence and Resistance in Presence of BCAs

### 2.1 BCAs, Their Modes of Action and the Acquisition of Resistance to These Antibiotics

BCAs comprise the three main groups, quinolones, ß-lactams and aminoglycosides.

Quinolones, especially fluoroquinolones, are meanwhile the most important antibiotics. The fluoroquinolones, active against Gram-negative and Gram-positive bacteria, represent the second generation of the synthetic quinolone antibiotics. Medically most widely used quinolone antibiotics are ciprofloxacin, norfloxacin, levofloxacin and some others. The quinolones interfere directly with DNA replication in bacteria by blocking the activity of the two type II topoisomerases, DNA gyrase and topoisomerase IV. Both proteins are essential for DNA replication by relaxing the positive supercoiled DNA formed ahead of the replication fork, and by decatenating the two DNA rings formed at the replication termination point, respectively. This is achieved by introducing transient double-strand breaks in phosphodiester bonds and rejoining them in an ATP-dependent reaction. The active DNA gyrase is composed of two subunits, GyrA and GyrB, forming an A_2_B_2_ complex. The supercoiling activity is mediated by the GyrA subunits, while the GyrB subunits are responsible for the ATPase activity required for supercoiling. The topoisomerase IV is also an A_2_B_2_ tetramer composed of the subunits ParC and ParE. For further details, see (Hooper, 2001; Heisig, 2009; Aldred et al., 2014; Hooper and Jacoby, 2016; Nitiss et al., 2019; Bush et al., 2020).

Bacteria acquire resistance to (fluoro)quinolones mainly by mutations in the chromosomal genes *gyrA* or *parC*, but also by several plasmid-determined gene products, such as Qnr and the aminoglycoside acetyltransferase AAC(6´)-Ib (Robicsek et al., 2006; Bush et al., 2020). The Qnr family members are characterized as pentapeptide proteins (PRPs) which probably mimic DNA and thus prevent binding of quinolones to gyrase and topoisomerase IV, whereas AAC(6´)-Ib modifies the (fluoro)quinolones resulting in reduction of its target-binding activity. Moreover, several efflux pumps can reduce the cellular concentration of quinolones. The import of quinolones depends on the density of porins in the outer membrane (OmpA and OmpX) (Woodford and Ellington, 2007; Minarini and Darini, 2012; Aldred et al., 2014; Hooper and Jacoby, 2015; Samadpour and Merrikh, 2018; Bush et al., 2020).

The other class of gyrase inhibitors is represented by the aminocoumarins which include the widely used drugs novobiocin and coumermycin. These antibiotics inhibit DNA gyrase - in contrast to quinolones - by binding to the ATPase active site of the GyrB subunit (Schröder et al., 2013; Heide, 2014). Because of serious side effects in patients, this group of gyrase inhibitors is now restricted to veterinary medicine.

β-Lactams, including penicillin and derivatives, cephalosporins, monobactams, carbapenems and carbacephems inhibit the last step in the synthesis of the peptidoglycan layer of bacterial cell walls through acylation of the transpeptidase which is involved in cross-linking of the different peptide moieties of Gram-positive and Gram-negative bacteria. This step is essential for the rigidity of peptidoglycan. The primary targets of the ß-lactams are the penicillin-binding proteins (PBPs). The interaction inhibits the terminal transpeptidation process and induces loss of cell viability and lysis by various autolytic processes. For further details, see  (Fisher and Mobashery, 2020).

Resistance to ß-lactam antibiotics, which is wide-spread and represents a severe health problem, is mainly caused by a large number of different ß-lactamases. These enzymes hydrolyze the ß-lactam ring common to all ß-lactam antibiotics thereby forming a linear metabolite incapable of PBP binding. The genes coding for the ß-lactamases are frequently localized on mobile genetic elements such as plasmids and transposons (Salverda et al., 2010), and can therefore be easily transmitted to other still sensitive bacteria by horizontal gene transfer (HGT). Point mutations in the ß-lactamase genes leading to amino acid changes in the ß-lactamases result in rapidly growing families of Extended Spectrum Beta-Lactamases (ESBLs) and TEM-ß-lactamases. For more details, in particular of ESBLs and metallo-β-lactamases, see (Bradford, 2001; Behzadi et al., 2020).

Other mechanisms causing resistance to ß-lactam antibiotics include alterations of the PBP targets (e. g. in *S. pneumoniae*), reduced access to the periplasm (mainly in Gram-negative bacteria, e.g. OprD of *P. aeruginosa*), or efflux of the antibiotics from the periplasm of the latter bacteria by specific pumps (Nikaido, 1985; Tang et al., 2014; Decousser et al., 2017; Fisher and Mobashery, 2020). Methicillin-resistance of *S. aureus* (MRSA) is acquired by uptake of the mobile Staphylococcal Cassette Chromosomal mec Element” (SCCmec) carrying the *mec*A or *mec*C gene encoding PB2a, a penicillin-binding protein with low affinity to customary ß-lactam antibiotics (Peacock and Paterson, 2015).

Aminoglycosides are the third group of BCAs active against a wide spectrum of Gram-positive and Gram-negative bacteria by affecting translation (Krause et al., 2016). Streptomycin, isolated from *Streptomyces griseus*, was the first representative of the meanwhile large group of aminoglycosides that include natural products from *Streptomyces* spp. (e.g., neomycin, kanamycin, tobramycin) and *Micromonospora* (e.g., gentamicin, sisomicin) as well as chemically modified derivatives of the natural aminoglycosides (e.g., amikacin, netilmicin, arbekacin, plazomicin). Aminoglycosides have been most frequently used for the treatment of serious bacterial infections for many decades. However, for several reasons (parenteral application, ototoxic and nephrotoxic effects), aminoglycosides are used more frequently for the treatment of severe infections in combination with ß-lactam antibiotics (Wright et al., 2017). Since the 1980s, these antibiotics were increasingly replaced by the new generation of cephalosporins, carbapenems, and fluoroquinolones which seem to be less toxic and recognize an even broader spectrum of bacterial pathogens than the aminoglycosides. Nevertheless, the development of novel aminoglycosides, such as arbekacin and plazomicin, designed to overcome the common aminoglycoside resistance mechanisms, has renewed the interest for the aminoglycosides (Cox et al., 2018).

Resistance to aminoglycosides is caused by several mechanisms: (i) enzymatic inactivation of the aminoglycosides by N-acetyltransferases (catalyzing acetyl-CoA-dependent acetylation of an amino group), O-adenyltransferases (catalyzing ATP-dependent adenylation of a hydroxy group), or phosphotransferases (catalyzing ATP-dependent phosphorylation of a hydroxy group); the responsible enzymes are found in Gram-positive and Gram-negative bacteria as well (Ramirez and Tolmasky, 2010), (ii) modifications of the 30S ribosomal subunit inhibiting the binding of the aminoglycosides, (iii) point mutations in the gene *fus*A1, which encodes the elongation factor G (EF-G1A) of *P. aeruginosa*, resulting in lower aminoglycoside affinity to the ribosome, (iv) decreased permeability of the bacterial cell membrane for these antibiotics (PMF-dependent import), in particular under anaerobic growth conditions, and (v) increased efflux of the antibiotics from the bacterial cell. For further details, see (Magnet and Blanchard, 2005; Wilson, 2014; Serio et al., 2017; Bolard et al., 2018).

### 2.2 Increased Production of Reactive Oxygen Species in Presence of BCAs

In addition to the well-studied primary targets of the BCAs, these drugs also lead to diverse, lesser known downstream events that contribute strongly to the killing effect. For a recent review on this topic, see (Baquero and Levin, 2021). A common feature observed for all BCAs appears to be – similar to other stress conditions – the increased production of ROS (Kohanski et al., 2007; Zhao and Drlica, 2014; Van Acker and Coenye, 2017; Hong et al., 2019). Despite previous challenging reports (Keren et al., 2013; Liu and Imlay, 2013), now there is rather general agreement that ROS, induced by BCAs, substantially contribute to the cell killing caused by these drugs (Zhao et al., 2015; Hong et al., 2019). However, the extent of ROS production and its contribution to the BCA-mediated cell killing seem to strongly depend on the environmental conditions, such as availability of oxygen and free ferrous/ferric iron (McBee et al., 2017; Van Acker and Coenye, 2017).

ROS are mainly generated in the electron transfer respiratory chain (ETC) by single-electron reduction of oxygen (Cabiscol et al., 2000; Imlay, 2003) and include superoxide anion (O_2_^-^), hydrogen peroxide (H_2_O_2_), and hydroxyl radicals (OH*). The latter radical is produced from H_2_O_2_ by the Fenton reaction catalyzed by Fe^2+^ (Henle and Linn, 1997; Cabiscol et al., 2000). O_2_^-^ has a short lifespan and a low diffusion rate across membranes, in contrast to H_2_O_2_ which has a long lifespan and a high diffusion rate across membranes; OH* has a very short lifespan. Hot spots for ROS production are the NADH dehydrogenase (NDH) and the succinate dehydrogenase (SDH) belonging to complex I and complex II, respectively, of the ETC (Messner and Imlay, 2002; Esterhazy et al., 2008). Aspartate oxidase (NadB) is another significant source for H_2_O_2_ production (and thus for ROS) in *E. coli* under aerobic conditions (Korshunov and Imlay, 2010). These ROS-generating enzymes are wide-spread among the bacterial pathogens (**Table 1**). It is notable that most bacterial pathogens showing a high frequency of mutation-based ABR (highlighted in yellow in **Table 1**) have these ROS-generating enzymes while those showing a low frequency (highlighted in blue in **Table 1**) lack these enzymes. This will also be further discussed later in another context.

**Table 1:** Bacterial pathogens and their enzymes and pathways influencing the cellular level of ROS.

A										
EBP	Genome Size (Mbp)	G+C content of chromosomal DNA	NAD(P)H-producing enzymes of the TCA-cycle			ROS-generating enzymes			Oxidative stress regulon	
		ca. %	IDH	OGDH	MDH	NDH	SDH	NadB	OxyR	SoxRS
eco	4,60	51	+	+	+	+	+	+	+	+
yen	4,55					+	+	+	+	+
ype	4,65					+	+	+	-	-
kpn	5,50	57	+	+	+	+	+	+	+	+
smar	4,90-6,30	59	+	+	+	+	+	+	+	+
vch	4,03					+	+	+	+	+
bpe	4,12					+	+	-	(+)	-
ngo	2,23	52	+	+	-	+	+	+	(+)	-
nme	2,27					+	+	+	(+)	-
efa	3,00					+	-	-	-	-
pae	5,60	67	+	+	-	+	+	+	+	(-)
abau	3,98	39	+	+	+	+	+	+	(+)	-
hpy	1,66	39	+	-	-	+	-	-	-	-
cje	1,64-1,90					+	+	-	-	-
tpa	1,14	52	-	-	-	-	-	-	-	-
*without a peptidoglycan layer*										
mpn	0,82					-	-	-	-	-
										
**B**										
bsu	4,21					+	+	+	-	-
ban	5,50					+	+	+	-	-
cdl	4,30					+	-	+	-	-
sau	2,80	33	+	+	-	+	+	-	-	-
spn	2,14	40	-	-	-	-	-	-	-	-
spy	1,85					-	-	-	-	-
										
**C**										
**IBP**										
*Vacuolar*										
sty (f)	4,60	52	+	+	+	+	+	+	+	+
lpn (f)	3,40					+	+	+	+	+
mtu (f)	4,40	65	+	+	+	+	+	+	-	-/-
bhe (f)	1,84					(-)	+	-	-	-/-
bme (f)	3,30					+	+	-	(+)*	-/-
cbu (o)	1,97					+	+	+	+	+
ctr (o)	1,00	41	-	+	+	-	-	-	-	-/-
Cytosolic										
sfl (f)	4,50	51	+	+	+	+	+	-	+	+
lmo (f)	2,90					-	-	+	-	-/-
ftu (o)	1,90	33	+	+	+	+	+	+	+	+
rpr (o)	1,10	29	+	+	+	+	+	-	-	-/-

abau, Acinetobacter baumanii; ban, Bacillus anthracis; bhe, Bartonella henselae; bme, Brucella melitensis; bpe, Bordetalla pertussis; bsu, Bacillus subtilis; cbu, Coxiella burnetii; cdl, Clostridioides difficile; cje, Campylobacter jejuni; ctr, Chlamydia trachomatis; efa, Enterococcus faecalis; eco, Escherichia coli; ftu, Francisella tularensis; hpy, Helicobacter pylori; kpn, Klebsiella pneumoniae; lmo, Listeria monocytogenes; lpn, Legionella pneumophila; mpn, Mycoplasma pneumoniae; mtu, Mycobacterium tuberculosis; ngo, Neisseria gonorrhoeae, nme, Neisseria meningitidis; pae, Pseudomonas aeruginosa; rpr, Rickettsia prowazekii; sau, Staphylococcus aureus; sfl, Shigella flexneri; smar, Serratia marcescens; spn, Streptococcus pneumoniae; spy, Streptococcus pyogenes; sty, Salmonella enterica ser. Typhimurium; tpa, Treponema pallidum; vch, Vibrio cholerae; Yen, Yersinia enterocolitica; ype, Yersinia pestis. (A) Extracellular Gram-negative bacterial pathogens (EBP); (B) Gram-positive EBP; (C) facultative intracellular (f) and obligate intracellular (o) bacterial pathogens (IBP), replicating preferentially in vacuoles or in the cytosol of their host cells. Pink underlined pathogens show high incidences of ABR (according to WHO), while blue underlined pathogens show low incidences of ABR.

Under normal growth conditions, bacteria keep the cellular levels of ROS in balance by several mechanisms: the superoxide dismutases, SodA and SodB, convert O_2_^-^ to H_2_O_2_, which is further detoxified by catalases (Cat) and alkyl hydroperoxide reductases (Ahp). There is no known enzyme that detoxifies cellular OH*. When the capacity of the constitutively expressed Sod and Cat enzymes is exhausted, the SoxRS and OxyR regulons of *E. coli* are induced by O_2_^-^ and H_2_O_2_, respectively (Christman et al., 1989; Pomposiello and Demple, 2001; Blanchard et al., 2007). This leads to enhanced gene expression of two regulons, the products of which prevent further increase of ROS (mainly by the induced Sod and Cat enzymes), or protect and repair structures damaged by ROS (Zheng et al., 1999; Pomposiello et al., 2001; Hebrard et al., 2009). Nevertheless, overproduction of ROS (in particular of OH*) leads to severe damage of essential cell components, particularly of proteins, lipids and DNA (Droge, 2002; Baquero and Levin, 2021).

In summary, the three major BCA groups, i.e. quinolones, β-lactams and aminoglycosides, apparently stimulate the production of ROS in oxygen-respiring bacteria. ROS seem to contribute significantly to the lethal effect of the BCAs by interfering with several essential cell structures, in particular DNA (Imlay, 2006; Dwyer et al., 2007; Kohanski et al., 2008; Hong et al., 2019; Rasouly and Nudler, 2019). Although these secondary ROS-dependent damages are caused by all bactericidal drugs, the ROS-dependent secondary damage seems to have a drug-specific context as well (Hong et al., 2019).

### 2.3 DNA Damage Generated by ROS and Induction of the SOS Response

In the following, we mainly focus on the ROS-triggered DNA lesions and on the subsequently induced SOS response as these events are crucially linked to ABR. The DNA lesions are caused in different ways by oxidation of nucleobases, mainly through OH*. DNA exposed to OH* yields more than 20 different oxidatively modified purine and pyrimidine bases (Demple and Harrison, 1994). Formation of 8-oxo-7,8-dihydroguanine (8-OxoG) by oxidation of guanine is the most abundantly altered base.

Incorporation of 8-OxoG in DNA can occur *via* direct oxidation of G in DNA or *via* oxidation of dGTP in the nucleotide pool to 8-OxodGTP, followed by incorporation of 8-OxodGTP into the DNA by DNA polymerase(s). MutT phosphatase (if present in the bacteria, see **Table 2**) can, however, sanitize the 8-OxoGTP pool by hydrolyzing it to 8-OxoGMP (Fowler and Schaaper, 1997). 8-OxoG incorporated into DNA is able to pair with both cytosine (C) and adenine (A) with an almost equal efficiency leading to a G→T transversion during replication or DNA repair (Sekiguchi and Tsuzuki, 2002; Mundt et al., 2008; Cerchiaro et al., 2009).

**Table 2:** EBPs and IBPs and their regulons and pathways involved in DNA repair.

A															
EBP	Genome Size	SOS Response		COM	NER			BER					TLS		
	(Mbp)	LexA	RecA	ComEC	UvrA	UvrB	UvrC	Ung	MutM	MutY	Nth (Endo III)	MutT	Pol II	Pol IV	Pol V
eco	4,60	+	+	+	+	+	+	+	+	+	+	+	+	+	+
yen	4,55	+	+	+	+	+	+	+	+	+	+	+	+	+	+
ype	4,65	+	+	+	+	+	+	+	+	+	+	+	+	+	-
kpn	5,50	+	+	+	+	+	+	+	+	+	+	+	+	+	+
smar	4,90-6,30	+	+	+	+	+	+	+	+	+	+	+	+	+	+
vch	4,03	+	+	+	+	+	+	+	+	+	+	+	+	+	-
bpe	4,12	+	+	-	+	+	+	+	+	+	+	+	-	+	-
ngo	2,23	-	+	+	+	+	+	+	+	+	+	-	-	+	-
nme	2,27	-	+	+	+	+	+	+	+	+	+	+	-	+	-
efa	3,00	+	+	+	+	+	+	+	+	+	+	+	-	+	-
pae	5,60	+	+	+	+	+	+	+	+	+	+	+	+	+	+
abau	3,98	+	+	+	+	+	+	+	+	+	+	+	-	+	+
hpy	1,66	-	+	+	+	+	+	+	-	+	+	-	-	-	-
cje	1,64-1,90	-	+	+	+	+	+	+	-	+	+	-	-	-	-
tpa	1,14	-	+	comE	+	+	+	-	-	+	+	-	-	-	-
*without a peptidoglycan layer*															
mpn	0,82	-	+	-	+	+	+	+	+	-	-	-	-	+	-
														
**B**															
bsu	4,21	+	+	+	+	+	+	+	+	+	+	+	-	+	Pol IV,2; +*
ban	5,50	+	+	+	-	+	+	+	+	+	+	+	-	+	+*
cdl	4,30	+	+	-	+	+	+	+	+	+	+	+	-	+	-
sau	2,80	+	+	+	+	+	+	+	+	+	+	+	-	+	+*
spn	2,14	-	+	+	+	+	+	+	+	+	+	+	-	+	-
spy	1,85	-	+	+	+	+	+	+	+	+	+	+	-	+	-
														
**C**															
**IBP**															
*Vacuolar*															
sty (f)	4,60	+	+	+	+	+	+	+	+	+	+	+	+	+	+
lpn (f)	3,40	UmuD*	+	+	+	+	+	(+)	+	+	+	+	–	+	+
mtu (f)	4,40	+	+	-	+	+	+	+	+	+	+	+	-	+	+
bhe (f)	1,84	+	+	+	+	+	+	(+)	+	+	+	+	–	+	+
bme (f)	3,30	+	+	(+)	+	+	+	–	+	+	+	+	–	+	–
cbu (o)	1,97	–	+	–	+	+	+	+	–	+	+	+	–	+	–
ctr (o)	1,00	-	+	-	+	+	+	+	-	+	+	-	-	-	-
*Cytosolic*															
sfl (f)	4,50	+	+	-	+	+	+	+	+	+	+	+	+	+	+
lmo (f)	2,90	+	+	+	+	+	+	+	+	+	+	–	–	+	–
ftu (o)	1,90	-	+	-	+	+	+	+	+	-	+	+	-	-	-
rpr (o)	1,10	-	+	-	+	+	+	-	-	-	+	-	-	-	-

COM, competence pathway; NER, nucleotide excision repair; BER, base excision repair; TLS, translesion DNA synthesis. For further abbreviations and color marking see legend to **Table 1** and text. Using Kegg´s Genes Data Base, the components involved in the major DNA repair pathways were screened for the most frequently occurring extra- and intracellular bacterial pathogens.

Additional DNA damage caused by ROS includes the formation of abasic sites and of single- and double-strand breaks (Chakarov et al., 2014) by stalling of the replication fork when it encounters ROS-generated obstacles (Radman, 1975; Marnef et al., 2017). These events are in part lethal (especially double-strand breaks) and in part mutagenic (e.g. single-strand breaks and base modifications), if not properly repaired by error-free repair processes (Girard and Boiteux, 1997; Pages and Fuchs, 2002).

Many bacteria react to ROS-generated DNA lesions by inducing the SOS response or/and other stress responses (Friedberg et al., 2006a; Wu et al., 2012; Maslowska et al., 2019). However, in all bacteria with a SOS regulon (**Table 1**), its induction appears to be the most important response to DNA damage.

In short, the SOS regulon is a tightly controlled network comprising more than 40 genes in *E. coli* (Simmons et al., 2008) that are engaged in cell protection and repair of essential cell structures (DNA, proteins, lipids). For more detailed information about the genes of bacterial SOS regulons, see (Kreuzer, 2013). The key regulators controlling this regulon are LexA and RecA (**Table 2**). Under unstressed growth conditions, dimeric LexA represses the transcription of all genes belonging to the SOS regulon by binding to the SOS-box, a specific sequence present in the promoter region of the genes belonging to the SOS regulon (Walker, 1984).

RecA is a co‐protease that stimulates self‐cleavage of LexA. RecA binds to single-stranded DNA and is thereby converted - in the presence of (d)ATP - to the active form (the RecA* nucleoprotein filament) that stimulates the self‐cleavage of LexA. This process decreases the LexA affinity for the SOS boxes leading to the gradual de-repression of SOS genes, depending on the binding strength of LexA to the respective SOS box of the LexA-controlled gene (Little and Mount, 1982; Neher et al., 2003).

Different DNA lesion events can generate ss-DNA, e.g. stalled replication caused by BCA-induced ROS, or HGT by conjugation or transformation (see below). These events can activate RecA, subsequently leading to induction of SOS response (Cox, 2003).

Increased BCA-triggered production of ROS in treated bacteria causing induction of RecA/LexA-dependent SOS response has been observed for all three groups of BCA under laboratory cultivation conditions (Phillips et al., 1987; Miller et al., 2004; Dörr et al., 2009; Baharoglu et al., 2014). Even ß-lactams, the primary targets of which are penicillin-binding proteins, i.e. extracellular proteins, affect - besides causing bacteriolysis - also respiration in *E. coli* and other bacteria leading to increased intracellular accumulation of ROS (Dwyer et al., 2015; Leger et al., 2019). Thus, ß-lactams can also induce in *E. coli* and other bacteria, similar as quinolones, an SOS response through activation *via* RecA/LexA (Kohanski et al., 2007; Kohanski et al., 2010; Dwyer et al., 2015).

Aminoglycosides also induce SOS response in several Gram-negative pathogens, like *Vibrio cholerae, Klebsiella pneumoniae, Photorhabdus luminescens*, but not in *E. coli* (Baharoglu et al., 2013). *E. coli* seems to restore DNA lesions formed by aminoglycosides preferentially by the SOS-independent very short patch mismatch repair (VSPR) and by mismatch repair (MMR) when VSPR is impaired (Baharoglu et al., 2014).

The SOS regulon also contains several genes whose products are directly involved in DNA repair (Baharoglu and Mazel, 2014; Maslowska et al., 2019). For our present discussion, the role of these SOS-induced DNA repair functions is highly relevant as some of these processes can subsequently lead to increased mutation rates and thus eventually to mutation-based ABR (Fuchs and Fujii, 2013).

The major DNA repair pathways to be considered are: (i) the nucleotide excision repair (NER), (ii) the base excision repair (BER), and in particular (iii) translesion DNA synthesis (TLS). The gene products encoded by bacterial SOS regulons that directly participate in DNA repair, include: UvrA and UvrB involved in the NER pathway and RecA, that - among other functions - also participates in the activation of a TLS DNA polymerase.

The TLS DNA polymerases are named (in *E. coli*) Pol II (product of *dinA*), Pol IV (product of *dinB*) and Pol V (or UmuD’_2_C; product of *umuC* and *umuD*). Pol IV and Pol V belong to the Y-family polymerases (Yang, 2014) and function with significantly higher error frequencies than Pol II as they lack the error-correcting exonuclease function which is inherent in Pol II and in the replicative polymerases Pol III and Pol I. Therefore, Pol IV and in particular Pol V cannot correct errors during the ongoing replication; such errors can be repaired only post-replicatively as a consequence of the SOS response. Pol V but not Pol IV depends on RecA for its activation (Goodman and Woodgate, 2013).

NER and BER are wide-spread DNA repair pathways among bacteria including bacterial pathogens (**Table 2**). Both repair systems work largely error-free; they are involved in the repair of different DNA lesions, i.e. NER is responsible for the removal of bulky DNA lesions (e.g. those induced by UV light), whereas BER takes care of individual base modifications (in particular those induced by oxidation, alkylation and deamination of single purines or pyrimidines, respectively (Wozniak and Simmons, 2022).

TLS is the most important pathway for post-replication repair showing high lesion tolerance (Chatterjee and Walker, 2017; Henrikus et al., 2018; Yang and Gao, 2018; Joseph and Badrinarayanan, 2020) at the expense of increased replication errors and hence high mutation rates. TLS appears to be therefore more relevant for the generation of mutation-based ABR and will be therefore described in some more detail in the following.

In contrast to the replicative DNA Pol III, the TLS DNA polymerases involved in post-replication repair of damaged DNA are capable of bypassing lesions in the template DNA. In *E. coli* and other bacteria (including some IBPs, see **Table 2**), three SOS-inducible DNA polymerases, Pol II, Pol IV (also named DinB), and Pol V (UmuD’_2_C) participate in TLS. In contrast to Pol II (which is a member of the high-fidelity B-family DNA polymerases), Pol IV and especially Pol V have rather low base selection fidelity. Both polymerases belong to the Y-family DNA polymerases which lack proof-reading activity (Goodman and Woodgate, 2013; Yang, 2014).

In *E. coli*, the TLS polymerases replace the replicative Pol III at DNA lesions with different efficiencies (Raychaudhury and Basu, 2011) and mainly act at stalled replication forks or in lesion-containing gaps left behind the replisome (Henrikus et al., 2018; Marians, 2018). Mutations performed in each of the genes encoding the three TLS polymerases suggest that Pol V is most efficient in bypassing DNA lesions. In addition, Pol V, but not Pol IV, can also participate in gap-filling reactions over several hundred nucleotides. Pol V interacts with the active RecA*-filament which seems to have a chaperone-like function for this TLS polymerase (Goodman and Woodgate, 2013; Fujii and Fuchs, 2020). Pol IV is most widespread among bacterial pathogens, while Pol II is found only within species of the family of *Enterobacteriaceae* and a few other Gram-negative enteric bacterial pathogens. PolV and PolV-related repair polymerases which give rise to the highest mutation rates (Maor-Shoshani et al., 2000), are less common among bacterial pathogens than Pol IV (**Table 2**).

### 2.4 DNA Repair in the Absence of LexA-Controlled SOS Regulons

Considering that a number of extra- and intracellular bacterial pathogens lacks LexA (**Table 2**) and hence a LexA-controlled SOS regulon, it is not surprising that there are also LexA-independent mechanisms which trigger DNA repair pathways to restore DNA damage generated by BCAs.

Quinolones can stimulate SOS-independent induction of RecA-mediated processes including HRR (Drlica and Zhao, 1997; López et al., 2007). SOS-independent induction of DNA repair pathways appear to be mainly responsible for the repair of aminoglycoside-caused DNA lesions, especially in Gram-positive pathogens (Claverys et al., 2006). Several *β*-lactams can induce the TLS polymerase IV (DinB) in a LexA/RecA-independent way (Pérez-Capilla et al., 2005). DNA repair genes of *Mycobacterium tuberculosis* can be also induced by BCA-triggered DNA damage in a LexA/RecA-independent manner (Rand et al., 2003). In *E. coli*, fluoroquinolones stimulate DNA damage repair functions by intra- and inter-chromosomal recombination through a LexA-independent mechanism (López et al., 2007).

As shown for *S. pneumoniae* which lacks LexA and the SOS regulon, aminoglycosides (as well as quinolones) lead instead to expression of the competence regulon (Com) which may replace the SOS regulatory network, and induces expression of *recA* and other DNA repair genes - in addition to the *com* genes essential for the transformation process (Martin et al., 1995; Prudhomme et al., 2006; Slager et al., 2014). Indeed, it has been argued that competence may have evolved as a DNA damage response in SOS-deficient bacteria (Charpentier et al., 2011).

Under stress conditions, *H. pylori*, also lacking an SOS regulon, can induce its DNA uptake machinery and an enzyme that liberates DNA from neighboring cells. This genetic exchange mechanism enhances recombination between exogenous DNA and the inherent genome. This process can repair DNA lesions in the recipient strain, but also significantly contributes to the high genetic diversity of *H. pylori* isolates and the high spread of antibiotic resistance among clinical strains (Dorer et al., 2010; Ailloud et al., 2021).

Among the intracellular bacteria, the obligate intracellular pathogen *Coxiella burnetii* resides within a unique vacuole with lysosomal characteristics (Hackstadt and Williams, 1981; Voth and Heinzen, 2007) where it is exposed to a variety of DNA damaging agents, including ROS (Akporiaye et al., 1983; Brennan et al., 2004). Compared to the other obligate IBPs, *C. burnetii* has more DNA repair genes (**Table 2**) that partly belong to a SOS regulon which is constitutively expressed due to the lack of LexA. This LexA-independent SOS arrangement seems to reflect a unique repair adaptation of *C. burnetii* to its hostile niche (Mertens et al., 2008).

In summary, BCAs stimulate production of ROS in oxygen-respiring bacteria (Dwyer et al., 2007). These highly deleterious molecules interfere with essential cell structures, including DNA (Imlay, 2006; Dwyer et al., 2007; Kohanski et al., 2007; Kohanski et al., 2008). Especially OH* cause DNA lesions which subsequently lead to the induction of SOS-dependent and SOS-independent error-free and, at increasing DNA damage error-prone DNA repair pathways (Imlay and Linn, 1987; Modell et al., 2014; Tan et al., 2015). The finding that the antibiotic-tolerant persister subpopulation of a bacterial culture apparently produces much less hydroxy radicals and forms less lethal DNA lesions than the major part of the population, which is killed by the antibiotics (Kim et al., 2011), is also in line with these conclusions.

Note that most bacterial pathogens with a high frequency of mutation-based ABR possess SOS or Com regulons encoding error-prone repair enzymes (in particular Y-type TLS polymerases), while those showing a low frequency lack SOS or Com regulons and the error-prone repair enzymes (**Table 3**). This will be discussed later in more detail below in another context.

**Table 3:** List of bacterial pathogens with the highest and lowest incidences of ABR (according to WHO).

**BP**	**Genome Size (Mbp)**	**G+C content of chromosomal DNA**	**TLS Polymeras es**			
		**ca. %**	**Pol II**	**Pol IV**	**Pol V**	**Others**
**A**						
eco	4,60	51	+	+	+	
kpn	5,50	57	+	+	+	
smar	4,90-6,30	59	+	+	+	
ngo	2,23	52	-	+	-	
efa	3,00	39	-	+	+++*	
pae	5,60	67	+	+	-	+
abau	3,98	39	-	+	+	
hpy	1,66	39	-	-	-	+ (Pol I)
sau	2,80	33	-	+	-	+
spn	2,14	40	-	+	-	
sty	4,60	52	+	+	+	
mtu	4,40	65	-	+	-	+
sfl	4,50	51	+	+	+	
**B**						
tpa	1,14	52	-	-	-	-
ctr	1,00	41	-	-	-	-
ftu	1,90	33	-	-	-	-
rpr	1,10	29	-	-	-	-

**(A)** Bacterial pathogens (BP) with the highest frequency of ABR; (yellow): priority 1, (green): priority 2, (blue): priority 3; **(B)** Bacterial pathogens with low frequency of ABR. For abbreviations, see legend to **Table 1** and text.

### 2.5 BCA-Induced DNA Repair can Lead to Increased Mutation Rates

The above described repair processes (particularly those carried out by the TLS polymerases of the Y-family) appear to be mainly responsible for the enhanced mutational events observed in presence of BCAs. The enhanced mutation frequency contributes to the remarkable ability of bacteria to rapidly adapt to these and other antibiotics and finally leads to the development of mutation-based ABR (Friedberg et al., 2006b; Kohanski et al., 2010; MacPhee and Ambrose, 2010; Baharoglu et al., 2013; Handel et al., 2015; Windels et al., 2019).

In particular, the ROS-induced generation of 8-OxoG frequently mispairs with adenine, leading to G→T transversion during DNA replication and DNA repair. This mispairing plays an essential role in increased mutagenesis and for the emergence of ABR observed upon treatment with BCAs (Kohanski et al., 2007; Kreuzer, 2013; Belenky et al., 2015; Kawai et al., 2015).

As described above, the SOS regulon comprises (among others) several genes encoding error-prone repair enzymes, in particular the TLS polymerases Pol IV and Pol V. These DNA polymerases are able – in contrast to the replicating Pol III – to overcome stalled replication sites, however, at the expense of increased mismatch base pairing leading to enhanced mutations. Among the three *E. coli* TLS polymerases (Pol II, IV and V), the mutation rate is highest for Pol V and lowest for Pol II (Fujii and Fuchs, 2020). In particular, the error-prone gap-filling activity of Pol V significantly contributes to the increased mutation frequency.

The induction of the TLS polymerases is timely controlled by the strength of the LexA-binding sites determining the expression of the operons encoding these polymerases. The *umuDC* operon encoding Pol V has one of the tightest LexA-binding sites and is induced last in the SOS response, i.e. 30–40 min after DNA damage (Sommer et al., 1993). The induction of the TLS DNA polymerases and, hence, the mutation frequencies will therefore depend on the cellular ROS concentration triggered by the BCAs.

The connection between BCA treatment, ROS production and increased mutagenesis is further suggested by the observation that bacteriostatic antibiotics, recognizing the same primary targets as BCAs, do not trigger ROS production and apparently cause fewer mutations and less mutational antibiotic resistance (Kohanski et al., 2007).

When severe DNA damage is sustained, activation of error-prone TLS DNA polymerase(s) can result in a further increased mutation rate. This is called the SOS mutator phenotype (or hypermutation) which strongly contributes to mutation-based ABR (Jolivet-Gougeon et al., 2011; Weigand and Sundin, 2012). In clinical context, such hypermutator strains of *P. aeruginosa* have been isolated from the lungs of cystic fibrosis (CF) patients and analyzed phenotypically by focusing on ABR and virulence and by genome sequencing to identify mutations associated with ABR development and metabolic adaptation to the environment of the respiratory tract of CF-patients (Hogardt et al., 2007; Woodford and Ellington, 2007; Colque et al., 2020). These studies of *P. aeruginosa* isolates of long-term-infection revealed novel regulatory pathways of genes involved in *amp*C expression (encoding the dominant ß-lactamase AmpC of *P. aeruginosa*), the regulation of more than six efflux pumps to diverse BCAs, and a mutation of *fus*A1 (encoding EF-G1 elongation factor) which inhibits aminoglycoside-binding to the ribosome (Lister et al., 2009; Poole, 2011; Lopez-Causape et al., 2018; Colque et al., 2020; Glen and Lamont, 2021; Zwama and Nishino, 2021).

### 2.6 Horizontal Gene Transfer can Also Induce SOS Response and Hence DNA Repair and Mutagenesis

As mentioned above, HGT, in particular conjugation and transformation, is the most important mechanism for intra- and inter-species DNA transfer in bacteria, including the transfer of antibiotic resistance genes. DNA transfer from donor to recipient bacterial cells by conjugation or transformation - even if the transfer event is abortive - generates single-stranded (ss) DNA. This ss-DNA can activate RecA and trigger a RecA*/LexA-induced SOS response in the recipient cell. As a result, expression of the genes encoding DNA repair enzymes is induced with the possible consequence of enhanced mutagenesis and the emergence of ABR (Baharoglu et al., 2010; Baharoglu et al., 2012; Liu et al., 2019; Virolle et al., 2020). However, this does not apply for all plasmid incompatibility (Inc) groups. The most frequently encountered plasmids in human infections (IncF, IncI and IncN plasmids) carry plasmid-encoded SOS-inhibitors such as PsiB (interacting with RecA) and the ssDNA-binding protein SSP which together suppress the SOS response in the donor cell (Golub et al., 1988; Petrova et al., 2009; Baharoglu et al., 2010). These SOS-response suppressors are pre-transferred to the recipient by the plasmid-encoded type IV secretion system (T4SS) of the donor and can even be produced by the recipient cell during ssDNA transfer (Virolle et al., 2020; Al Mamun et al., 2021). Integrases encoded by integrons, if present in the recipient bacteria, can also be up-regulated during this process resulting in increased rearrangements of these chromosomal cassettes (Baharoglu et al., 2010).

In addition, induction of SOS response due to BCAs (e.g. ß-lactams, fluoroquinolones)-triggered DNA damage can induce, in suitable donor bacteria (e.g. *V. cholera, Salmonella, S. aureus*), the expression of genes necessary for the conjugative transfer. As a result, the transfer frequency of plasmids, transposons, integrons and lysogenic phages carrying ABR genes (and eventually pathogenicity islands) from the donor to recipient bacteria can be enhanced (Beaber et al., 2004; Hastings et al., 2004; Maiques et al., 2006; Bearson and Brunelle, 2015; Liu et al., 2017a; Blazquez et al., 2018).

It should be emphasized that HGT-linked events may also play a crucial role in triggering the spreading of mutation-based ABR. A typical example is *N. gonorrhoeae* that has developed severe resistance to many antibiotics (Tapsall, 2005; Unemo and Shafer, 2014) (**Table 2**). This ABR has been mainly developed by spontaneous mutations and transfer of the mutant genes to other sensitive gonococcal strains. *N. gonorrheoae* has a high capability for HGT and, therefore, ABR gene(s) arising by mutation in any strain can easily be transferred to other gonococcal strains. The antibiotic resistant strains are then further selected and distributed by antibiotic pressure in the clinics and by social contacts. Such events are not only a major driving force for HGT of antibiotics resistance genes in clinical and social connections, but can also enforce the spread of ABR genes between bacteria in the environment (Davies, 1994; Martinez, 2008; Lopatkin et al., 2016). However, conjugational plasmid transfer requires energy (ATP and PMF) and hence declines in the stationary growth phase and upon starvation (Palmen et al., 1994; Headd and Bradford, 2018; Neil et al., 2021).

In this context, it is worth mentioning that even non-antibiotic anti-microbial chemicals, such as certain heavy metal ions, including Cu^2+^, Ag^+^, Cr^6+^, and Zn^2+^ at environmentally-relevant concentrations (Zhang et al., 2018) and even antimicrobial agents, like triclosan, widely used in consumer products such as toothpaste, skin creams, deodorants, soaps and plastics (Halden and Paull, 2005), can promote the transfer of ABR genes within and across bacterial genera in the environment (Lu et al., 2018). The mechanisms of this phenomenon seem to involve again enhanced ROS formation with the subsequent induction of SOS response, leading to increased expression of SOS-controlled genes which are essential for the activation of TLS polymerases, and of conjugation-relevant genes in the donor (Lu et al., 2018; Zhang et al., 2018).

In summary, HGT stimulated by BCAs can enhance the intra- and interspecies transfer of preexisting ABR plasmids, transposons and integrons. Thereby, it also significantly contributes to the generation and spreading of new ABR genes.

### 2.7 Persister Formation is Enhanced by BCAs

Similar to other stress conditions, the treatment with BCAs leads in growing bacterial populations to enhanced formation of antibiotic-tolerant persister subpopulations (Gollan et al., 2019; Eisenreich et al., 2020; Zou et al., 2020). Indeed, the phenomenon of bacterial persistence was first reported in staphylococcal infections treated with penicillin (Bigger, 1944). Treatment of bacterial cultures with BCA is meanwhile the most frequently used method in studies analyzing persister formation in model bacteria (mainly *E. coli*).

The data obtained in these studies clearly show that the treatment of an antibiotic-sensitive bacterial population with members of all three BCA groups does not only select already existing, stochastically generated persister cells, but triggers, particularly when the antibiotic is applied at sub-inhibitory concentrations, the formation of antibiotic-insensitive persisters (Cui et al., 2018; Li et al., 2018). The persister subpopulation created by a single BCA is often also tolerant to other antibiotics, including bacteriostatic ones (Keren et al., 2012; Li et al., 2018).

Thus, it is reasonable to assume that in the BCA-generated persistence state the bacterial cell metabolism is altered such that persister cells in general avoid the lethal downstream processes induced by all BCAs, even when they recognize different primary targets. As mentioned above, increased ROS production appears to be a common downstream process apparently triggered by all BCAs. Among other cell damages, ROS lead to DNA lesions, which in turn trigger induction of the SOS regulon and, hence, of DNA repair pathways. This step appears to be also important for persister formation: mutants that lack the *lexA* or the *recA* gene and which are therefore unable to induce the SOS regulon are (i) more susceptible to BCAs, such as (fluoro)quinolones, and, (ii) exhibit significantly reduced persistence in presence of the BCAs (Dörr et al., 2009; Fung et al., 2010; Wu et al., 2012). On the other hand, constitutive expression of *lexA* and *recA* strongly enhances persistence upon treatment with these antibiotics (Dörr et al., 2009). These results suggest that persistence triggered by BCAs requires the ability of the bacterial cell to repair DNA damage. In line with this hypothesis, the key role of the SOS response in the generation of bacterial persister cells has been recently shown for several clinically significant bacterial pathogens (Podlesek and Zgur Bertok, 2020).

The metabolic background of the persister cells, in which they are refractory to multiple antibiotics, is still poorly defined. However, the existing data show that the dormant” state associated with persistence is characterized by low energy production and reduced anabolic metabolism, including slowed-down synthesis of essential macromolecules (Lewis, 2007; Amato et al., 2014; Harms et al., 2016).

ROS production, induced by BCAs, and the associated killing events, especially the extended DNA lesions, are apparently reduced to a lower level in the persistence state (Grant et al., 2012; Trastoy et al., 2018). As described above, ROS are generated mainly in the ETC, especially by complex I NADH dehydrogenase (NDH) and complex II succinate dehydrogenase (SDH). NDH and SDH normally use quinones (ubiquinone, menaquinone) as electron acceptors under aerobic conditions. However, if the electrons are accidentally transferred to oxygen, ROS, particularly O_2_^-^ and subsequently H_2_O_2_ and OH*, can be produced (see above). Slowing-down of NADH-yielding reactions in the TCA cycle, in particular those that are catalyzed by α-ketoglutarate dehydrogenase and malate dehydrogenase has been observed in the persister state. The resulting reduction of cellular NADH levels probably leads to a reduced production of ROS followed by less ROS-induced cell killing – an essential prerequisite for persister formation (Trastoy et al., 2018; Jung et al., 2019). Furthermore, fumarate reductase, if present, is induced under oxygen-limited conditions (e.g. in biofilms and in the stationary phase) and converts - opposite to SDH - quinols (mainly reduced menaquinone) to quinones using fumarate as electron acceptor. This respiratory process occurs without generating ROS and may also contribute to persister formation (Kim et al., 2016). These metabolic changes probably also lead to the induction of most factors and pathways outlined above which have been shown to stimulate persister formation.

The shut-down of the primary targets recognized by the BCAs (DNA replication, protein and cell wall synthesis, respectively), together with these downstream metabolic changes caused by BCAs, inhibit (directly or indirectly) *de novo* protein synthesis and ATP production. This in turn prevents the formation of the strictly regulated ATP-DnaA complex which together with additional proteins, including DiaA and IHF, is required for the initiation of new rounds of DNA replication and cell division (Leonard and Grimwade, 2010; Katayama et al., 2017; Hansen and Atlung, 2018). As already mentioned above, we recently hypothesized that the blockade of this step might be the final and decisive step for the establishment of the persistent state in bacteria (Eisenreich et al., 2020).

In this context, it should not go unmentioned that bacteriostatic antibiotics, e.g. tetracyclines, which do not induce synthesis of ROS, can also trigger persister formation by yet largely unknown mechanisms. Altered expression of DNA repair pathways, arrested protein synthesis and various other processes have been suggested at least in part as cause for the persister formation under these conditions (Kwan et al., 2013; Cui et al., 2018). Our proposed ATP-DnaA/OriC-based hypothesis can also readily explain the persister formation in the presence of bacteriostatic antibiotics (Eisenreich et al., 2020).

### 2.8. The Frequency of Mutations in General and, in Particular, of Mutation-Based ABR Is Increased in the Persistence State

A considerable number of reports show that the bacterial persistence state also promotes the generation of genetically fixed ABR (Cohen et al., 2013; Vogwill et al., 2016; Jung et al., 2019; Levin-Reisman et al., 2019; Liu et al., 2019; Windels et al., 2019; Bakkeren et al., 2020). This is rather unexpected, since both traits represent - as described above - entirely different mechanisms to cope with antibiotics.

As described above, BCAs can trigger production of ROS that damage DNA in a number of ways. These events induce the expression of error-free and error-prone DNA repair pathways by SOS response. Especially the error-prone repair mechanisms enhance the mutation frequency in general and can thereby also lead to increased mutation-based ABR within the persistent subpopulations (Vogwill et al., 2016; Sebastian et al., 2017; Yang and Walsh, 2017; Levin-Reisman et al., 2019; Windels et al., 2019).

According to our hypothesis, the state of persistence is characterized by the terminated DNA replication phase. In this phase, most bacterial genomes are in a closed circular supercoiled form that could still contain not yet repaired incorrect bases, such as 8-OxoG, as well as abasic sites, but no lethal double-stranded breaks which are mainly formed during the replication elongation step. As discussed above, it can be assumed that the persister cell contains a reduced ROS level and probably active SOS-induced repair pathways, including TLS (Dörr et al., 2009).

TLS is a post-replication repair pathway which uses as main tools error-prone DNA polymerases, i.e. Pol IV and Pol V in *E. coli* and related enteric bacteria (**Table 2**) or other Pol V-related Y-family DNA polymerases especially in Gram-positive bacteria (**Table 2**) (Timinskas and Venclovas, 2019). DNA lesions, such as incorrect base pairings, abasic sites, can still be present or even newly generated by ROS in the terminated DNA of the persisters. These lesions can be repaired especially by the TLS DNA polymerases, however, at the expense of increased mutations. The thereby increased chromosomal mutation rate will also lead to mutations that cause resistance to the challenging antibiotic and even to other antibiotics.

A common property of all DNA repair pathways, including TLS, is their dependence on ATP. This means that repair of DNA lesions can only occur as long as a residual energy metabolism is maintained in the damaged cell. ATP deficiency has serious consequences when DNA damage occurs in the elongation phase of DNA replication as this can lead to lethal double strand breaks that can be no more repaired. Residual ATP supply is also necessary for the viability of persister cells (Völzing and Brynildsen, 2015). Even in persister cells with terminated DNA replication, DNA damage has to be repaired before proper re-initiation of DNA replication can occur. In the complete absence of ATP, even persister cells are unable to awake.

Whether spread of ABR, determined by conjugative plasmids, can also occur through horizontal gene transfer (HGT) in the persistence state remains an open question. Studies on mice perorally infected with *Salmonella* Typhimurium showed that, after treatment with ceftriaxone, the persister population surviving in intestinal tissue is, when released into the gut lumen, still capable of promiscuous conjugation and can foster the spread of plasmids in the gut, including ABR plasmids (Bakkeren et al., 2019). However, this study does not show, whether transfer of the plasmids already occurred when the donor salmonellae were still in the persister state (the persister state of intracellular salmonellae is based on the action of t-RNA acetyltransferase TacT and leads to a partial blockade of translation, probably without harmful loss of ATP (Rycroft et al., 2018) or – more likely – already in the awakened state.

Based on our (still speculative) hypothesis on the generation of the bacterial persistence state (Eisenreich et al., 2020), we assume that - in principle - persister cells could act as donors for the transfer of conjugative plasmids even in the persistence state under certain conditions. According to this hypothesis, the lack of a functional ATP-DnaA/oriC complex is the ultimate cause for the generation of the persistence state. Transfer of conjugative plasmids is initiated at the transfer origin (oriT) and is independent of ATP-DnaA. Conjugation initiated at oriT requires a plasmid-encoded relaxase which introduces a single strand break in oriT (Lee and Grossman, 2007). The transfer of the formed single strand plasmid DNA across the membrane needs ATP and PMF (proton motive force) (Palmen et al., 1994). This requirement could be met in the persisters if the lack of functioning ATP-DnaA/oriC complex is due to a blockade of protein synthesis or to a missing new oriC site in the persister donor cells rather than due to the lack of ATP. As noted above, the expression of the plasmid-encoded Tra proteins, also required for the transfer of a conjugative plasmid to a bacterial recipient cell (Lee and Grossman, 2007), is even induced in the persistence state.

Taken together, the state of persistence may not only lead to increased mutation-based ABR due to the enhanced mutation rate, but could even allow the spread of ABR plasmids (if present in the persister bacteria) to suitable recipients in the environment.

## 3 The Susceptibility to Mutation-Based ABR Appears To Be linked to the presence of error-prone DNA repair pathways

An interesting concept, explaining the presence or absence of DNA repair pathways, termed proteomic constraint”, has been described by Acosta and colleagues (Acosta et al., 2015). They postulated that the number of DNA repair pathways, which a bacterial species carries, positively correlates with the information content of its genome (called the *proteome value P*).

This concept seems to apply among extracellular bacterial pathogens for *H. pylori*, *C. jejuni* and *Treponema pallidum* (**Table 2**) and especially for the obligate IBPs, i.e. *C. trachomatis, F. tularensis* and *R. prowazeki* (**Table 2**). These pathogens lack genes for LexA and many of the SOS-controlled DNA repair enzymes, in particular TLS polymerases, whereas genes for the NER-associated enzymes UvrA, B and C and RecA (involved in HRR) are present. As pointed out above, an exception is *C. burnetii* which lives within the host cell in a particularly hostile niche and is more threatened by host cell generated ROS. This obligate IBP has developed a LexA-independent SOS regulon and has more DNA repair functions than the other obligate IBPs, e.g. *C. trachomatis* and *R. prowazekii* (Mertens et al., 2008) (**Table 2**).

There seems to be also a correlation between the susceptibility to develop ABR and the presence of DNA repair pathways (especially of the error-prone TLS) among the bacterial pathogens (**Table 2**). This applies in particular to the IBPs. There are also several extracellular bacterial pathogens with relatively small genomes, e.g. *N. gonorrhoeae*, *S. aureus*, *S. pyogenes*, *S. pneumoniae*, and *Enterobacter* spp., that are equipped with most DNA repair genes (**Table 2**), including genes for TLS polymerases that appear to be particularly responsible for high mutation rates and, therefore, for the emergence of mutation-based ABR (Joseph and Badrinarayanan, 2020). As mentioned above, these pathogens also show a high propensity for natural transformation (*N. gonorrhoeae, S. pyogenes, S. pneumoniae*) or transduction (*S. aureus*) which may further enhance their susceptibility to ABR.

These correlations are also consistent with the data of the WHO Global Priority List (for 2017 and 2020) which includes multidrug resistant bacteria that pose a particular threat in hospitals (**Table 3**). The bacterial pathogens in the most critical group of community- or hospital-acquired infections (including *Acinetobacter baumanii*, *P. aeruginosa* and various *Enterobacteriaceae* spp., in particular *K. pneumoniae* and uropathogenic *E. coli*) have become resistant to a large number of antibiotics, including carbapenems and third generation cephalosporins, that represent the best available antibiotics for treating multidrug resistant (MDR) bacteria. Most bacterial pathogens of this group possess all properties that – as described above – favor the development of mutation-based ABR: (i) the ETC enzymes (NDH/complex I and SDH/complex II) and NadB, generating ROS under oxidative stress conditions, (ii) NAD(P)H-delivering enzymes of the TCA cycle, necessary for the function of complex II (SDH) (**Table 1**), (iii) the SOS and/or Com regulons, and (iv) error-prone repair enzymes, in particular TLS DNA polymerases belonging to the Y-family (**Table 2**) (Tang et al., 2000; Tegova et al., 2004; Norton et al., 2013; Aranda et al., 2014; Bunnell et al., 2017). Exceptions to this rule are *H. pylori* and *S. pneumoniae* which lack most of these properties. But as stated above, their high frequency of mutation-based ABR is favored by their high propensity for natural transformation competence.

*Vice versa*, lower mutation rates and lower susceptibility to ABR are exclusively observed in (mainly obligate intracellular) bacterial pathogens that have low proteome values p (see above) and in particular lack all error-prone TLS polymerases (**Table 3**) (Rolain and Raoult, 2005; Stamm, 2015; Mestrovic and Ljubin-Sternak, 2018). However, the members of this latter group are still able to efficiently form persisters in the presence of BCAs (Eisenreich et al., 2020). This indicates that the state of persistence alone is not enough to trigger increased mutation-based ABR. Rather, it seems to be the combination of persistence and the presence of active error-prone DNA repair pathways which is decisive for the increased formation of ABR in the presence of BCAs (Mo et al., 2016).

Obviously, intracellular bacterial pathogens (IBPs), when growing within mammalian host cells, are rarely if at all in contact with unrelated bacteria. Thus, transfer of ABR *via* HGT is rather unlikely under intracellular conditions. However, under these conditions, the IBPs are exposed to ROS produced by the host cells, especially by phagocytes. The concentration of ROS, in general detrimental for the IBP, depends on the particular host cell and the host cell niche that the IBP occupies (Dupre-Crochet et al., 2013). The oxygen and nitrogen radicals generated by the host cells (e.g. H_2_O_2_, NO, HOCl) can also lead to lesions in the bacterial DNA and subsequently to the induction of stress responses, e.g. induction of SOS response (if present), with increased mutation rates and, as a consequence, the generation of mutation-based ABR.

From the data shown in **Tables 1**, **2**, it is obvious that facultative IBPs (e.g. *S. enterica* Typhimurium, *S. flexneri*, and *M. tuberculosis*) show a much higher rate of ABR than obligate IBPs (e.g. *C. trachomatis* and *R. prowazekii*). This also applies to antibiotics that can reach the interior of the host cells. Some of the facultative IBPs even belong to the bacterial pathogens with the highest susceptibility to develop ABR, while most of the bacterial pathogens with the lowest susceptibility to ABR are obligate IBPs. Nevertheless, these latter pathogens, especially several *Chlamydia* species, are the causative agents of common infectious diseases in humans and are treated with antibiotics (including BCAs) similar to those used for infections caused by facultative IBPs. There are two main reasons for the different incidences of ABR in facultative and obligate IBPs:

(i) Most of the facultative IBPs (e.g. *Salmonella enterica* in broiler house) are efficient recipients (and subsequent donors) of transmissible ABR plasmids (Martinez and Baquero, 2002; Schlüter et al., 2007). In contrast, obligate IBPs carry less often transmissible plasmids and rarely serve as recipients of such plasmids (Bordenstein and Reznikoff, 2005; Sandoz and Rockey, 2010; Borel et al., 2016). In addition, the spread of these plasmids to and from facultative IBPs generally occurs in the extracellular state and in pathogen-specific favorable environments, e.g. the gut for enteric IBPs, where they can encounter potential donors of conjugative ABR plasmids (Wain et al., 2003; Baker et al., 2018; Puzari et al., 2018; Bakkeren et al., 2019; McMillan et al., 2020). Obviously, these opportunities of acquiring and spreading ABR are less accessible to obligate IBPs. Natural environments, as well as terrestrial or marine mammals, may also serve as reservoirs for the spreading of ABR plasmids by HGT to and from facultative IBPs, e.g. *Listeria monocytogenes*, *L. pneumophila*, and *Brucella melitensis* (Charpentier and Courvalin, 1999; Wattam et al., 2009; Allen et al., 2010; Von Wintersdorff et al., 2016; Shevtsov et al., 2017; Kuang et al., 2018; Olaimat et al., 2018; Baquero et al., 2020). Stress conditions caused by reduced pH, reduced temperature and increased osmotic pressure - applied in food preservation - have also been shown to trigger the transfer of conjugative plasmid between pathogenic and nonpathogenic bacteria. In addition to HGT, exposure to these environmental stresses, as well as biofilm formation, and - connected to it - increased formation of persister cells, also favor the development of mutation-based ABR in facultative IBPs (Beuls et al., 2012; Olaimat et al., 2018).

(ii) The mutation rates and hence the acquisition of mutation-based ABR is considerably higher in facultative IBPs than in obligate IBPs (Woodford and Ellington, 2007; Gomes et al., 2016). Indeed, mutations are the main cause of ABR in some facultative IBPs, in particular *M. tuberculosis* (Gygli et al., 2017). Resistance to the antibiotics which are active against this pathogen, given the apparent lack of HGT (Lawrence, 1999; Gagneux and Small, 2007; Bolotin and Hershberg, 2015), appears to be mainly caused by chromosomal mutations (Gygli et al., 2017).

Resistance to synthetic antibiotics, e.g. fluoroquinolones, is also predominantly acquired by mutations. Mutation-based resistance against these antibiotics occurs in facultative IBPs much more frequently than in obligate IBPs (Almahmoud et al., 2009; Rakic-Martinez et al., 2011; Shadoud et al., 2015; Zhang et al., 2016; Cuypers et al., 2018; Deguchi et al., 2018; Siebert et al., 2019), although such mutants can be obtained even with obligate IBPs by *in vitro* mutagenesis (Binet and Maurelli, 2005; Rolain and Raoult, 2005; Biswas et al., 2006). The low frequency of mutation-based ABR in obligate IBPs is consistent with the fact that these bacteria (with the exception of *Coxiella* spp.) have less error-prone DNA repair functions (especially TLS) than facultative IBPs (**Table 2**), which - as discussed above - seem to be mainly responsible for high mutation rate and mutation-based ABR *in vivo* and especially in the persistence state.

The finding that the obligate IBP *C. burnetii* shows a higher susceptibility to ABR than *R. prowazekii, F. tularensis* and *C. trachomatis* (**Table 2**) also fits into this concept (Rolain and Raoult, 2005; Sandoz and Rockey, 2010; Vranakis et al., 2011; Caspar and Maurin, 2017; Mestrovic and Ljubin-Sternak, 2018; Vanrompay et al., 2018). Although *Chlamydia* and *Coxiella* species clearly differ in their susceptibility to ABR, they both have a similar high tendency to develop persistence (Beatty et al., 1995; Maurin and Raoult, 1999; Kazar, 2005; Panzetta et al., 2018; Eisenreich et al., 2020). This again demonstrates that it is not the state of persistence *per se* which is responsible for the generation of increased mutation-based ABR (Salcedo-Sora and Kell, 2020), but rather the combination of persister formation along with the ability to perform error-prone DNA repair.

**Figure 2:**
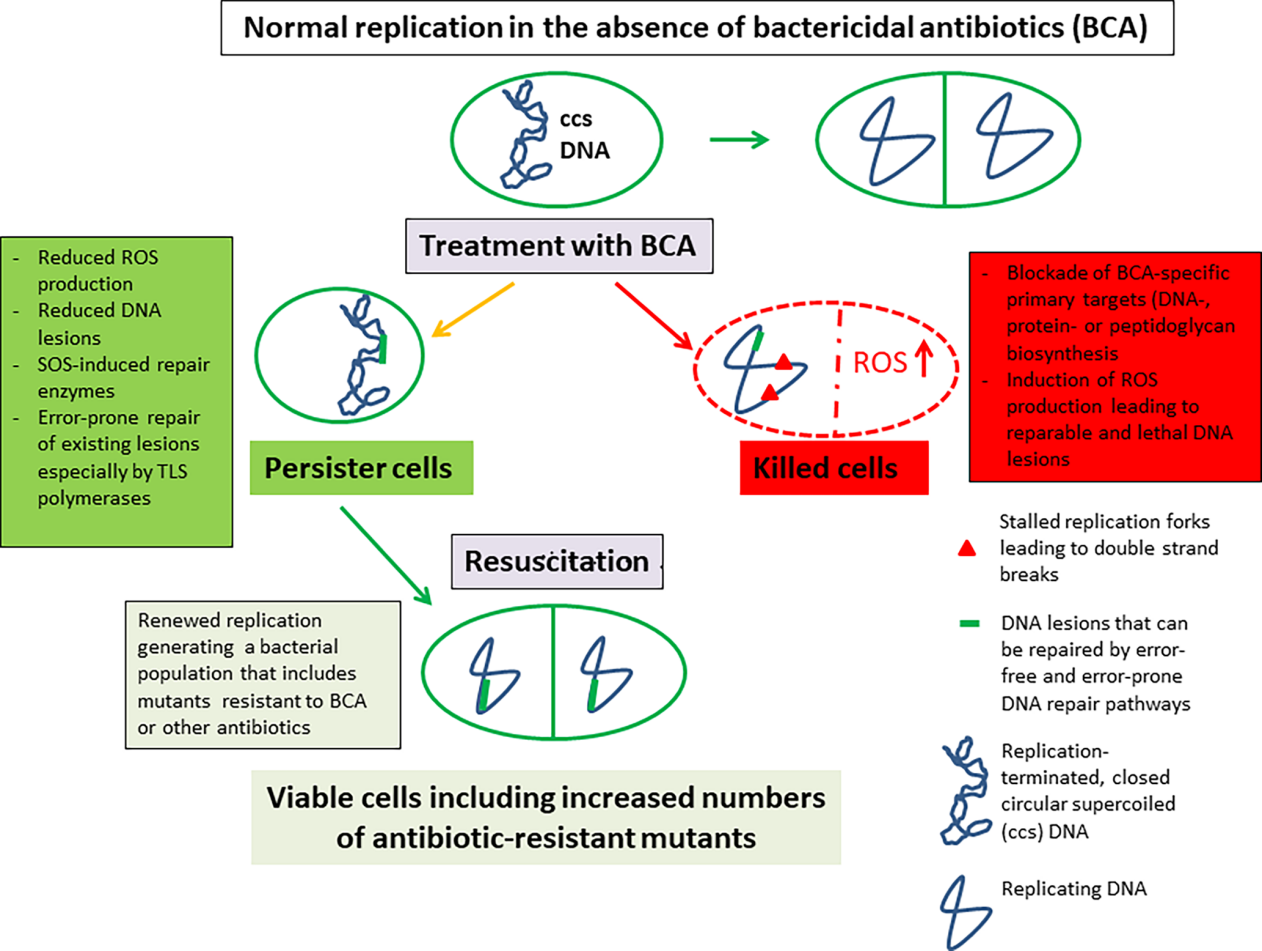
Emergence of mutation-based ABR in persisters. Treatment of a bacterial population with BCAs leads to killing of the majority of the bacterial cells that are in an active DNA replication phase and to the generation of persisters with terminated DNA replication (see Figure 2 and text for details). The ccs chromosomal DNA of the persisters may contain non-lethal DNA damages (green bars) that can be repaired mainly by error-prone DNA repair processes, e.g. by TLS DNA polymerases, however at the expense of increased mutation rates that lead – among others – to ABR mutants.

The persistence state is characterized – according to our hypothesis (Eisenreich et al., 2020) – by the inability of the bacteria to initiate a new round of DNA replication due to an insufficient amount of the ATP-DnaA initiator complex (**Figure 1**). In this state, the chromosomal DNA of most bacteria is in a closed circular supercoiled (ccs) form which is less susceptible to lethal DNA lesions, such as double strand breaks, than DNA that is still in the elongation process of replication. DNA lesions that can still be introduced by ROS into the terminated ccs DNA in the persistence state, such as nucleobase (especially guanine) oxidation and generation of abasic sites, can be repaired before or after resuscitation, depending on the cellular ATP concentration, by error-free and especially error-prone repair processes. However, the latter processes will give rise to increased mutations including mutations that lead to ABR (**Figure 2**).

It is also interesting to note that the chromosomal DNAs of the obligate IBPs, with low mutation-based ABR, have also a rather low G+C content (**Table 1**) and hence may show a lower susceptibility to G oxidation which leads to the formation of highly mutagenic 8-OxoG. It is in accord with this assumption that these obligate IBPs also lack MutY and/or MutT (**Table 2**), which remove 8-OxoG incorporated into the DNA and free 8-OxoGTP from the cellular GTP pool, respectively.

## 4 Summary and Conclusions

It is now widely accepted that treatment of bacterial pathogens with BCAs that block different key cellular processes, i.e. DNA replication, protein biosynthesis or cell wall synthesis, also leads to increased ROS generation by disturbed respiration and subsequently to induction of oxidative stress responses. In addition to other cell damage, ROS cause DNA lesions, the severity of which depends on the cellular ROS concentration. Intracellular bacterial pathogens (IBPs) may in addition be affected by ROS, produced by their respective (often phagocytic) host cells as defense against the invading bacteria.

These detrimental processes kill the vast majority of bacteria in a growing population. However, a subpopulation whose formation is apparently further triggered by the BCAs will enter the persistence state in which the bacteria are insensitive to these anti-bacterial processes. Although a large number of *in vitro* and animal model studies, in part outlined above, convincingly document the link between BCA treatment, ROS production and persister formation, it should not go unmentioned that its clinical significance (e.g. in chronic infections) is less clear and studies addressing this problem are still rather scarce (Fauvart et al., 2011; Fisher et al., 2017; Jung et al., 2019).

The persistent bacteria apparently carry out an altered, more anoxic metabolism characterized by reduced ATP and ROS synthesis. In the persistence state, the bacteria are tolerant to the challenging BCA and even to additional antibiotics, but remain genetically unaltered compared to the original bacterial population, and upon resuscitation, which occurs under favorable physiological conditions, these persisters become again antibiotic-sensitive (see **Figure 2**).

BCA-induced ROS lead in many bacteria to the induction of SOS- or Com-regulons. Both regulons include genes encoding also - in addition to the specific functions involved in oxidative stress response and competence, respectively - enzymes for error-free and error-prone repair of DNA lesions. These genes are timely induced depending on the severity of the DNA damage. The genes encoding the error-prone TLS DNA polymerases are induced last. Most of the TLS DNA polymerases in contrast to the replicative DNA polymerases (Pol III and Pol I) are able to pass through even severe DNA lesions – however at the expense of a high mutation rate (Joseph and Badrinarayanan, 2020).

According to our hypothesis (Eisenreich et al., 2020), the persistence state is characterized by the inability of the bacterial cell to initiate a new round of replication of its chromosomal DNA due to the lack of a sufficient amount of ATP-DnaA and hence of a functional orisome complex that is decisive for the re-initiation of DNA replication (Leonard et al., 2019). In this replication termination state, the chromosomal DNA is in a closed circular supercoiled (ccs) form (provided the chromosomal DNA is circular, which is the case in most bacterial pathogens). The ccs DNA is - compared to actively replicating circular DNA - rather insensitive to lethal DNA lesions, such as double strand breaks (Mizuuchi et al., 1980). Less severe (and repairable) DNA lesions, including those that arise from nucleobase (especially guanine) oxidation and the generation of abasic sites, can still be introduced by ROS (especially by hydroxyl radicals) into the ccs chromosomal DNA present in the persistence state. However, these lesions can be repaired before or immediately after resuscitation, mainly by error-prone repair processes, provided the cell contains still enough ATP to repair the lesions. These repair processes will, however, cause increased mutations, including mutations that lead to resistance against the challenging BCA and/or other antibiotics. Thus, the antibiotic-tolerant persistence state, together with the error-prone repair processes, can significantly enhance the emergence of genetically fixed (mutation-based) ABR.

The particular importance of the error-prone DNA repair processes - especially those carried out by the error-prone TLS DNA polymerases - is also suggested by the high frequency of mutation-based ABR in those bacterial pathogens that possess these enzymes and the significantly lower susceptibility to mutation-based ABR in other bacterial pathogens that lack the main error-prone repair enzymes.

These conclusions are supported by the fact that the bacterial pathogens with the highest incidence of ABR (according to WHO) are EBPs or facultative IBPs, which possess the NAD(P)H-delivering reactions of the TCA cycle and the enzymes of the ETC (and in most cases also NadB), that are involved in ROS production under aerobic conditions (**Tables 2**, **3**). These pathogens may acquire ABR mainly by ROS/persistence-induced mutagenesis. Obvious exceptions to this notion are *S. aureus, S. pneumoniae* and *H. pylori*. These pathogens gain their high incidence of ABR mainly by transformation (*H. pylori* and *S. pneumoniae*) or by transduction (*S. aureus*) and less by ROS/persistence-induced mutagenesis.

The insights reported in this review suggest that the problem of ABR could be reduced by the suppression of persister formation arising by antibiotic treatment. Based on the current knowledge concerning bacterial persister formation under different physiological stress conditions, several anti-persister strategies have already been proposed (Defraine et al., 2018). A promising therapy that emerges from our discussion could involve the inhibition of the bacteria-specific error-prone repair enzymes, especially of the Y-family TLS polymerases, by specific inhibitors (Yamanaka et al., 2017; Ketkar et al., 2019) to prevent mutations possibly also leading to ABR, and - after the antibiotic treatment - the inactivation of DnaA to prevent re-initiation of DNA replication and cell division of the generated persisters (Makise et al., 2002; Schenk et al., 2017; Samadpour and Merrikh, 2018; Grimwade and Leonard, 2019).

## Author Contributions

All authors wrote the review. The overall concept and design is based on an idea of WG. All authors contributed to the article and approved the submitted version.

## Funding

WE and TR thank the Deutsche Forschungsgemeinschaft for financial support (EI 384/16 and RU 631/17).

## Conflict of Interest

The authors declare that the research was conducted in the absence of any commercial or financial relationships that could be construed as a potential conflict of interest.

## Publisher’s Note

All claims expressed in this article are solely those of the authors and do not necessarily represent those of their affiliated organizations, or those of the publisher, the editors and the reviewers. Any product that may be evaluated in this article, or claim that may be made by its manufacturer, is not guaranteed or endorsed by the publisher.
